# Facteurs influençant l'initiation au traitement antirétroviral des personnes vivant avec le VIH dans les Centres de Traitement Agréés de Bamenda et de Bertoua au Cameroun

**DOI:** 10.11604/pamj.2014.17.6.3221

**Published:** 2014-01-13

**Authors:** Francois-Xavier Mbopi-Keou, Esther Voundi Voundi, Ginette Claude Mireille Kalla, Irène Emah, Fru Angwafo, Walinjom Muna

**Affiliations:** 1 Faculty of Medicine & Biomedical Sciences, University of Yaounde I, Yaounde, Cameroon; 2 National Public Health Laboratory, Ministry of Public Health, Yaounde, Cameroon; 3 University Teaching Hospital, Yaounde, Cameroon; 4 Gynecologic and Paediatric Hospital, Yaounde, Cameroon

**Keywords:** VIH, Personnes vivant avec le VIH, Traitement antirétroviral, Bamenda, Bertoua, Centre de traitement agrée, Cameroun, VIH, People living with HIV, antiretroviral treatment, Bamenda, Bertoua, approved treatment centers, Cameroun

## Abstract

**Introduction:**

L'objectif de ce travail était de déterminer les facteurs influençant l'initiation au traitement antirétroviral des personnes vivant avec le VIH (PVVIH) dans les centres de traitements agrées (CTA) de Bamenda et de Bertoua au Cameroun.

**Méthodes:**

Il s'agissait d'une étude transversale, analytique réalisée de Janvier à Avril 2011, dans les CTA de Bamenda et de Bertoua. Pour cette étude, nous avons obtenu une clairance éthique.

**Résultats:**

Nous avons étudiés 460 dossiers de patients séropositifs en phase d'initiation au traitement antirétroviral dans les CTA de Bamenda et de Bertoua, 53,9% et 46,1% respectivement. L ‘âge médian était de 36 ans. La plupart des séropositifs à Bertoua (41) avaient fait un dépistage volontaire du VIH par rapport à ceux de Bamenda (22) (p= 0.008). Il y ‘avait plus de VIH de type 1 et 2 dans le CTA de Bamenda (15) par rapport à Bertoua (3) (p= 0.011). La majorité des patients était classé au stade clinique II à Bamenda (54,0%) tandis qu ‘à Bertoua le stade clinique III était prédominant (52,4%) (p = 0,000). Le taux médian de CD4 était de 133 cellules/mm3 dans le CTA de Bamenda et de 175 cellules/mm3 à Bertoua (p = 0,008). La Zidovudine était plus prescrit à Bamenda et le Ténofovir à Bertoua (p = 0,000). L ‘Efavirenz était plus prescrit à Bertoua tandis que la Névirapine l ‘était plus à Bamenda (p = 0,000). Le Lopinavir/r était plus prescrit à Bamenda qu ‘à Bertoua (p = 0,017).

**Conclusion:**

Il apparait urgent de standardiser la prise en charge des PVVIH dans les CTA du Cameroun.

## Introduction

La pandémie de l ‘infection par le Virus de l'Immunodéficience Humaine (VIH) est loin d ‘être contrôlée et ce nonobstant les grandes interventions menées à l ‘échelle planétaire. L′Afrique subsaharienne abrite plus de 67% de toutes les Personnes Vivant avec le VIH (PVVIH), soit 22,5 millions [1]. L′accès au traitement antirétroviral (TAR) en Afrique subsaharienne reste très faible en raison d′obstacles [2–4] tels que le nombre limité de médecins, la mesure de la charge virale limitée, la problématique de la disponibilité d′examens plus simples comme la mesure des lymphocytes T CD4 ou les examens de biochimie [1]. Devant ces difficultés, l′Organisation Mondiale de la Santé (OMS) a proposé une approche de prise en charge «allégée» pour favoriser l′accès aux antirétroviraux (ARV) à grande échelle dans les pays à ressources limitées [5]. Les premiers programmes africains structurés d′accès aux ARV ont vu le jour en 1998 dans des pays comme le Sénégal, la Côte d′Ivoire et l′Ouganda [6, 7]. Ces programmes ont montré l′efficacité du TAR en Afrique, avec des résultats comparables à ceux obtenus dans les pays du Nord en termes de survie, d′efficacité virologique, immunologique et clinique, d′observance, d′émergence des résistances et de toxicité [9, 10].

Dès l ‘apparition des premiers cas de SIDA en 1985 au Cameroun [11], divers plans de lutte contre le VIH/SIDA ont été élaborés et mis en'uvre avec plus ou moins de succès de 1987 à nos jours [12]. Des progrès encourageants méritent d ‘être inscrits tels que l ‘accroissement important du nombre total des Centres de Traitement Agréés (CTA) et des Unités de Prise En charge du VIH/SIDA (UPEC), la gratuité des ARV depuis mai 2007, l ‘augmentation du nombre de personnes séropositives sous ARV [13]. Le Comité National de Lutte contre le SIDA (CNLS) rapporte en Décembre 2009 que sur 560 000 PVVIH, le Cameroun compterait près de 74.710 patients sous antirétroviraux (ARV), soit 39% des 153.185 PVVIH éligibles aux traitements dans l ‘ensemble du pays [14, 15].

Au regard de toutes ces améliorations dans la prise en charge des PVVIH, il s ‘avère indispensable de déterminer les facteurs influençant la mise sous traitement antirétroviral. Aussi, l ‘objet de cette étude était d ‘identifier les déterminants de l ‘initiation au traitement antirétroviral en comparant les caractéristiques sociodémographiques et biocliniques, le traitement antirétroviral des PVVIH des CTA des deux régions à plus forte prévalence du VIH au Cameroun, à savoir l ‘Est et le Nord-Ouest-Cameroun.

## Méthodes

**Schéma d ‘étude:** Il s'agissait d ‘une étude transversale, analytique réalisée sur une période de 4 mois, de Janvier à Avril 2011, dans les CTA des Hôpitaux Régionaux de Bertoua et de Bamenda au Cameroun.

**Population d ‘étude:** Etaient retenus dans notre étude, les patients séropositifs âgés de plus de 15 ans, en phase d ‘initiation de traitement, régulièrement suivi dans les deux CTA. Ceux transférés dans les CTA ou en échec thérapeutique y étaient exclus. Cette étude a fait l ‘objet d ‘une clairance éthique. A la fin de chaque comité thérapeutique, les informations étaient recueillies auprès des patients et dans leurs dossiers après avoir obtenu leur consentement. Le questionnaire utilisé s ‘intéressait aux caractéristiques sociodémographiques du patient, à la circonstance de découverte de son statut sérologique, à la durée entre le dépistage et l ‘initiation au TAR, à son entourage à savoir le nombre d ‘enfants, le statut sérologique des enfants de moins de 3 ans et pour les marié(e)s, le statut sérologique de leur(s) partenaire(s). Les dossiers permettaient de réunir les données sur le profil bioclinique du patient et le TAR choisi au cours du comité.

**Analyse des données:** Pour l'analyse des données, nous avons utilisé le logiciel EpiData Analysis Version 2.2.1.171. Les comparaisons entre les patients du CTA de Bamenda et ceux du CTA de Bertoua étaient faites à l ‘aide du test de Chi2 pour les variables qualitatives et du test ANOVA pour les variables quantitatives. Etait considérée comme différence statistiquement significative, une p value (p) <0,05.

## Résultats

**Distribution générale:** Nous avons recruté 460 patients séropositifs en phase d'initiation dans les CTA de Bamenda et de Bertoua, respectivement 53,9% et 46,1%. Cent cinquante huit séropositifs étaient de sexe masculin contre 302 de sexe féminin soit un sexe ratio de 1 homme pour 2 femmes (Tableau 1).

**Tableau 1:** Caractéristiques sociodémographiques des patients en phase d'initiation au traitement antirétroviral dans les centres de traitement agréés de Bamenda et de Bertoua au Cameroun

	CTA de Bamenda		CTA de Bertoua		p value
	n= 248	%	n = 212	%	
**Sexe**					0,11
Féminin	171	69,0	131	61,8	
Masculin	77	31	81	38,2	
**Statut matrimonial**					0,07
Célibataire	83	33,5	60	28,3	
Concubinage	3	1,2	11	5,2	
Divorcé(e)	19	7,7	12	5,7	
Marié(e)/monogamie	95	38,3	91	42,9	
Marié(e)/polygamie	7	2,8	10	4,7	
Veuf/Veuve	41	16,5	28	13,5	
**Lieu de résidence**					0,79
Hors région	12	4,8	10	4,7	
Région	101	40,7	80	37,7	
Ville	135	54,4	122	57,5	
**Type d'emploi**					0,000
Fonctionnaire	27	10,9	32	15,2	
Privé	147	59,1	75	35,5	
Sans emploi	74	30,0	105	49,3	
**Age (années)**					0,44
Minimum	17		16		
Médiane	36		35		
Maximum	76		66		

**Caractéristiques socio-démographiques:** L’âge médian était de 36 ans à Bamenda et de 35 ans à Bertoua et variait entre 17-76 ans et entre 16-66 ans respectivement (p = 0,44). Il y'avait plus de patients de sexe féminin autant dans le CTA de Bamenda (69,0%) que dans celui de Bertoua (61,8%). Le statut marié(e)-monogamie était le plus représenté dans les 2 CTA. Les patients séropositifs résidant dans les villes de Bamenda et de Bertoua étaient plus représentés, 54,4% et 57,5% respectivement. Le secteur privé était le plus représenté à Bamenda (59,1%) tandis que les sans-emploi l’étaient plus à Bertoua (49,3%) (p= 0.00) (Tableau 1).

**Statut sérologique:** Concernant la circonstance de dépistage, la majorité des patients avait soit découvert leur statut sérologique au cours d'une maladie (75,4% à Bamenda contre 68,4% à Bertoua), ou bien lors d'un dépistage volontaire (8,9% à Bamenda contre 19,3% à Bertoua) (p = 0,008). La durée entre le dépistage et l'initiation du traitement antirétroviral variait entre 0 et 96 mois avec une médiane de 1 mois dans le CTA de Bamenda et entre 0 et 90 mois avec une médiane de 2 mois au CTA de Bertoua (p = 0,499). Il y avait plus de VIH de type 1 et 2 dans le CTA de Bamenda par rapport à celui de Bertoua, 15 et 3 respectivement (p= 0.011). La majorité des patients était classé au stade clinique II à Bamenda (54,0%) tandis qu’à Bertoua, le stade clinique III était prédominant (52,4%) (Tableau 2) (p = 0,000). La plupart des patients étaient à un stade immunologique sévère à Bamenda (61,3%) et à Bertoua (55,7%) (p = 0,399) (Tableau 2).

**Tableau 2:** Données sur le statut sérologique et caractéristiques biocliniques des patients en phase d'initiation au traitement antirétroviral dans les centres de traitement agréés de Bamenda et de Bertoua au Cameroun

	CTA de Bamenda		CTA de Bertoua		p value
	n= 248	%	n = 212	%	
**Circonstance de découverte**					0,008
Maladie	187	75,4	145	68,4	
PTME	23	9,3	12	5,7	
Sujet contact	16	6,5	14	6,6	
Volontaire	22	8,9	41	19,3	
**Type de VIH**					0,011
Type 1	233	94,0	209	98,6	
Type 1&2	15	6,0	3	1,4	
**Stade clinique (OMS)**					0,000
Stade I	29	11,7	36	17,0	
Stade II	134	54,0	46	21,7	
Stade III	67	27,0	111	52,3	
Stade IV	18	7,3	19	9,0	
**Stade immunologique (OMS)**					0,399
Sévère	152	61,3	118	55,7	
Avancé	88	35,5	84	39,6	
Modéré	6	2,4	5	2,4	
Normal	2	0,8	5	2,4	
**Coinfection Tuberculose/VIH**					0,000
Oui	23	9,3	53	25,0	
Non	225	92,7	159	75,0	
**Coinfection Hépatite B/VIH**					0,013
Oui	10	2,8	1	0,5	
Non	238	97,2	211	99,5	
**Taux de CD4 (/mm** ^**3**^ **)**					0,008
Minimum	1		1		
Médiane	133		175		
Maximum	555		825		
**Durée entre dépistage et initiation (mois)**					0,499
Minimum	0		0		
Médiane	1		2		
Maximum	96		90		
**Poids actuel (Kg)**					0,000
Minimum	36		30		
Médiane	60		55		
Maximum	110		102		
Taux d'hémoglobine (g/dL)					0,000
Minimum	5,3		7,6		
Médiane	11,0		11,3		
Maximum	17,7		16,6		

**Entourage:** Cent quatre patients avaient des enfants de moins de 3 ans parmi lesquels 31 (29,8%) connaissaient leur statut sérologique. La majorité soit 72,7% à Bamenda et 70,0% à Bertoua était séronégatif (p = 0,87). Des 203 patients mariés, 105 (51,7%) connaissaient le statut sérologique de leur partenaire. La majorité soit 60,0% à Bamenda et 74,0% à Bertoua était séropositif (p = 0,13) (Tableau 3).

**Tableau 3:** Entourage des patients en phase d'initiation au traitement antirétroviral dans les centres de traitementagréés de Bamenda et de Bertoua au Cameroun

	CTA de Bamenda		CTA de Bertoua		p value
	n	%	n	%	
**Connaissance du statut sérologique**					
**Enfant < 3ans**					0,359
Oui	11	25,0	20	33,3	
Non	33	75,0	40	66,7	
**Partenaire(s)**					0,361
Oui	55	54,5	50	48,1	
Non	46	45,5	54	51,9	
**Statut sérologique**					
Enfant < 3ans					0,873
Négatif	8	72,7	14	70	
Positif	3	27,3	6	30	
Partenaire(s)					0,128
Négatif	22	40	13	26	
Positif	33	60	37	74	
TAR:					

**Caractéristiques biocliniques:** Le taux de CD4 variait entre 1 et 555 cellules/mm3 avec une médiane de 133 cellules/mm3 dans le CTA de Bamenda et entre 1 et 825 cellules/mm3 avec une médiane de 175 cellules/mm3 à Bertoua. Il y avait une différence statistiquement significative (p = 0,008). Le poids des patients variait entre 36 et 110 Kg avec une médiane de 60 Kg à Bamenda et entre 30 et 102 Kg avec une médiane de 55 Kg à Bertoua. Cette différence était statistiquement significative (p = 0,000). Il y avait plus de patients ayant la tuberculose dans le CTA de Bertoua (53 contre 23, p= 0.000) tandis qu'il y avait plus de patients ayant l'hépatite virale B dans le CTA de Bamenda (10 contre 1, p = 0,013). Le taux d'hémoglobine variait entre 5,3 et 17,7 g/dl avec une médiane de 11,0 g/dl dans le CTA de Bamenda et entre 7,6 et 16,6 g/dl avec une médiane de 11,3 g/dl à Bertoua (p = 0,000) (Tableau 2).

**Traitement antirétroviral:** La Zidovudine était plus prescrit à Bamenda et le Ténofovir à Bertoua (p = 0,000). L'Efavirenz était plus prescrit à Bertoua tandis que la Névirapine l’était plus à Bamenda (p = 0,000) (Tableau 4). L'utilisation du Lopinavir/r était plus marquée à Bamenda qu’à Bertoua (p = 0,017). Concernant la prescription des autres ARV, il n'y avait pas de différence statistiquement significative entre les deux CTA (Tableau 4).

**Tableau 4:** Prescription des antirétroviraux aux patients en phase d'initiation au traitement antirétroviral dans les centres de traitement agréés de Bamenda et de Bertoua au Cameroun

		CTA de Bamenda		CTA de Bertoua		p value
		n= 248	%	n= 212	%	
* INRT	Zidovudine	210	84,7	92	43,4	0,000
	Ténofovir	38	15,3	120	56,6	
* INRT	Lamivudine	235	94,8	204	96,2	0,452
	Emtricitabine	13	5,2	8	3,8	
** INNRT	Efavirenz	88	37,8	145	60,1	0,000
	Névirapine	145	62,2	83	39,9	
Lopinavir/r	Oui	16	6,5	4	1,9	0,017
	Non	232	93,5	208	98,1	
Abacavir	Oui	2	0,8	0	0	0,191
	Non	246	99,2	212	100	

* INRT: inhibiteur de la reverse transcriptase

** INNRT : inhibiteur non nucléosidique de la reverse transcriptase

## Discussion

Au cours des dernières années, la question de l’équité dans l'accès aux soins pour les personnes vivant avec le VIH/SIDA s'est posé, le risque étant que l'accès aux soins soit limité aux populations des zones plus développées [5, 6] telles que Bamenda par rapport à Bertoua. Cette étude nous a permis de d’étudier 460 dossiers de PVVIH. Parmi lesquels 53,9% étaient suivi au CTA de Bamenda et 46,1% à Bertoua, ceci pouvant s'expliquer par la démographie plus importante dans la ville de Bamenda [5, 6].

Les personnes vivant avec le VIH ayant participé à l'enquête étaient en majorité des femmes (302 sur 460) autant dans le CTA de Bamenda (69,0%) que dans celui de Bertoua (61,8%). Cette proportion élevée s'explique d'une part par la réalité épidémiologique de l’épidémie au Cameroun, plus de 60% de ces personnes sont des femmes [16] et, d'autre part, par une couverture en antirétroviraux plus élevée dans la population féminine [17]. L’âge médian était de 36 ans à Bamenda et de 35 ans à Bertoua et variait entre 17-76 ans et entre 16-66 ans respectivement. Des résultats similaires ont été retrouvés au cours de l'enquête EVAL-ANRS [17] où l’âge médian de la population étudiée était de 36,8 ans. Le secteur d'emploi des patients sous antirétroviraux suivis à Bamenda était majoritairement le secteur privé tandis qu’à Bertoua la plupart était sans emploi (49,3%). L'enquête EVAL-ANRS a révélé que la majorité des PVVIH exerçait dans le secteur informel (54,5%) au niveau régional [17]. Ces différences pourraient s'expliquer par le fait que la région de l'Est est la moins développée des deux en ce qui concerne le statut économique [17].

La raison du dépistage était préférentiellement au cours d'une maladie autant à Bamenda qu’à Bertoua (75,4% et 68,4%) mais plus de PVVIH faisaient leur dépistage volontairement à Bertoua qu’à Bamenda (19,3% contre 8,9%). La durée entre le dépistage et l'initiation au traitement antirétroviral était de 1 mois à Bamenda et de 2 mois à Bertoua, ce qui dénote des lacunes dans le dépistage précoce de l'infection à VIH et par conséquent de la sensibilisation.

Une faible proportion de patients suivis dans les 2 CTA était considérée comme coinfectée au VIH type 1 et 2, mais la majorité était du CTA de Bamenda (6% contre 1,4% à Bertoua). Le stade clinique III de l'OMS était plus présent dans le CTA de Bertoua et le stade clinique II à Bamenda. Paradoxalement, bien qu'au niveau de la classification immunologique de l'OMS il n'y avait pas de différence statistiquement significative (le stade sévère étant plus représenté dans les 2 CTA), le taux de CD4 médian dans le CTA de Bamenda était plus petit qu’à Bertoua au moment de l'initiation du traitement. Ce qui laisse sous-entendre une entrée plus précoce dans les soins chez les patients suivis à Bertoua. Par ailleurs, il est intéressant de noter que le poids des patients en phase d'initiation était plus élevé chez ceux suivis au CTA de Bamenda. Ceci pourrait s'expliquer par le fait que l'agriculture soit la principale activité dans la région du Nord-Ouest. Les traitements de première ligne contenant de la Zidovudine étaient plus souvent prescrits dans le CTA de Bamenda alors que le taux d'hémoglobine des patients en phase d'initiation était moins élevé chez eux. Ceci pourrait s'expliquer par les problèmes de rupture fréquente des ARV que les formations sanitaires connaissent.

## Conclusion

Les similitudes entre les patients séropositifs des deux CTA étaient l’âge, la prédominance féminine, celle du statut marié(e)-monogamique, de la ville comme lieu de résidence, de la maladie comme raison de dépistage du VIH, de la séronégativité des enfants de moins de trois ans, de la séropositivité des partenaires des couples mariés. Ces caractéristiques pourraient être considérées comme facteurs influençant la mise sous traitement antirétroviral. Néanmoins, des différences avaient été observées au niveau du type de VIH, du stade clinique, du taux de CD4, du taux d'hémoglobine, du poids actuel, des coinfections tuberculose et hépatite virale B. Concernant les traitements antirétroviraux, les ruptures de médicaments ainsi que la prédominance de certaines pathologies pourraient expliquer les différences observées. Notre étude mais surtout en exergue l'urgente nécessité de standardiser la prise en charge des PVVIH dans les CTA du Cameroun.
